# Quantitative Association Between Otorrhea Frequency and Sensorineural Hearing Risk in Pediatric Chronic Otitis Media

**DOI:** 10.3390/jcm15145653

**Published:** 2026-07-19

**Authors:** Yurino Nagata, Masaomi Motegi, Kazuaki Chikamatsu

**Affiliations:** Center for Advanced Hearing Implants and Hearing Aids, Gunma University Hospital, Department of Otolaryngology–Head and Neck Surgery, Gunma University Graduate School of Medicine, 3-39-15 Showamachi, Maebashi 371-8511, Gunma, Japan; y-nagata@gunma-u.ac.jp (Y.N.); tikamatu@gunma-u.ac.jp (K.C.)

**Keywords:** chronic otitis media, cholesteatoma, sensorineural hearing loss, otorrhea, pediatric, bone conduction, surgical indication, cochlear protection

## Abstract

**Background/Objectives**: Chronic otitis media (COM) in children carries a risk of irreversible sensorineural hearing loss (SNHL), which impairs language development and academic performance, although an objective clinical threshold does not exist to identify patients requiring urgent surgical intervention. **Methods**: We retrospectively analyzed 60 pediatric patients (aged 5–18 years) who underwent surgery for COM or cholesteatoma and performed linear regression, Pearson’s correlation, and receiver operating characteristic (ROC) curve analyses to quantify the relationship between otorrhea frequency and shifts in bone conduction (BC) threshold at least six months postoperatively. **Results**: Otorrhea frequency was significantly correlated with BC deterioration (*r* = 0.327, *p* = 0.011, *R^2^* = 0.107). ROC analysis identified five or more episodes as the optimal cutoff for predicting a clinically significant BC shift of ≥10 dB (area under the curve = 0.712; sensitivity 75.0%, specificity 80.0%). Bacteriological findings, including the presence of non-skin commensal pathogens, did not significantly influence hearing outcomes (*p* = 0.721). **Conclusions**: Five or more otorrhea episodes constitute a quantitative, equipment-free red flag for imminent cochlear injury in pediatric COM, and this threshold provides primary care physicians and pediatricians a simple, age-independent criteria for timely surgical referral to prevent lifelong auditory morbidity.

## 1. Introduction

Chronic otitis media (COM), which includes chronic suppurative otitis media and middle ear cholesteatoma, is a persistent inflammatory condition characterized by recurrent otorrhea and progressive hearing impairment. Although conductive hearing loss is common, persistent inflammation, which is often exacerbated by drug-resistant bacteria, can spread to the inner ear through the round window membrane. This process leads to sensorineural hearing loss (SNHL) due to irreversible damage to hair cells and the spiral ganglion [1]. Once SNHL is established, it often remains refractory to conventional treatments, resulting in a permanent decline in quality of life (QOL).

The implications of SNHL in the pediatric population are particularly significant. Even a mild elevation in bone conduction (BC) thresholds, especially in the high-frequency range, can significantly impair language development and academic performance [2,3]. Recent cross-sectional and cohort studies have demonstrated that children with COM exhibit elevated BC thresholds that do not improve even after the resolution of middle ear inflammation [4]. Notably, such sensorineural deterioration has been reported even in children under the age of five, emphasizing that the inner ear is vulnerable from a very early age.

Despite the well-established risk of cochlear injury, no objective criteria exist to guide the timing of surgical intervention for pediatric COM. Some clinicians advocate surgery before school age to optimize developmental outcomes [5], whereas others recommend delaying any intervention until the eustachian tube function matures or until the child can better tolerate postoperative care [6]. This lack of consensus stems from difficulties in identifying specific cases that are at the highest risk of developing irreversible SNHL. Therefore, a readily accessible clinical marker for quantifying this risk is urgently required.

Otorrhea is one of the most accessible clinical indicators of active middle ear inflammation. Although recurrent episodes of otorrhea are the clinical hallmarks of COM, their quantitative relationship with the progression and severity of SNHL in children has not been adequately established. If a clear correlation exists, the frequency of otorrhea could serve as a vital “red flag” for clinicians.

Clarifying these risks may justify early surgical intervention over conservative management in high-risk pediatric cases, assisting otorhinolaryngologists, pediatricians, and primary care physicians worldwide in preventing lifelong auditory morbidity. Therefore, this study aimed to investigate the quantitative relationship between otorrhea frequency and severity of SNHL in pediatric COM and to establish an objective clinical limit for BC threshold shifts that can inform surgical decision-making. By determining the clinical cutoff for otorrhea frequency that predicts significant BC threshold shifts, we aimed to provide objective evidence to support clinical decision-making.

## 2. Materials and Methods

### 2.1. Patients and Study Design

**Figure 1:**
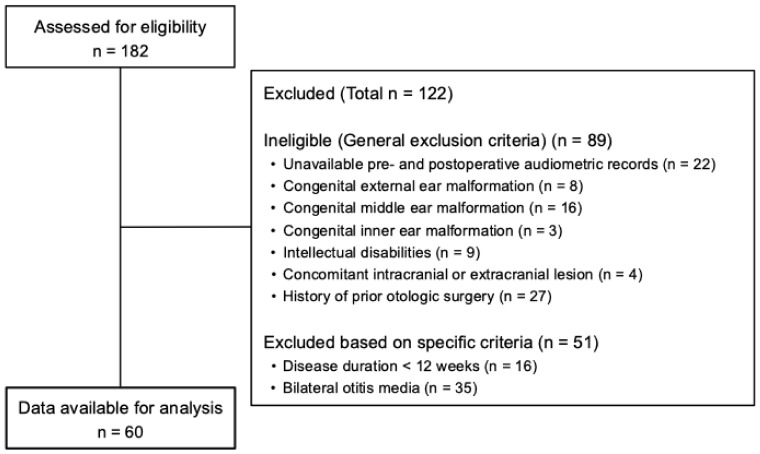
Flowchart of study subject inclusion. Of the 182 screened patients, 60 met the inclusion criteria and were included in the analysis. The primary reasons for exclusion were bilateral otitis media (*n* = 35) and a history of otologic surgery (*n* = 27).

This retrospective study was conducted at a university hospital. We reviewed the medical records of pediatric patients (aged 5–18 years) who underwent surgery for perforated tympanic membrane or middle ear cholesteatoma between 2008 and 2025. Patients with incomplete pre- or postoperative audiometric data were excluded. Consequently, the following exclusion criteria were applied based on the medical history and clinical findings: (1) hereditary hearing loss, (2) bilateral otitis media, (3) intracranial or extracranial complications, (4) congenital malformations of the external, middle, or inner ear, (5) intellectual disability, (6) a history of prior otologic surgery, (7) use of local or systemic ototoxic medication, (8) a history of meningitis or labyrinthitis, and (9) the presence of a labyrinth fistula. To focus on the chronic effects of the unilateral pathology, we excluded patients with bilateral disease. After applying these criteria, 60 patients (60 ears) were included in the final analysis (Figure 1). The study was conducted in accordance with the Declaration of Helsinki and was approved by the Institutional Review Board of the participating institution (protocol code: HS2023-241, 9 May 2024). Patient consent was waived due to the retrospective nature of the study, and an opt-out method was used to provide patients and their guardians with the opportunity to refuse participation.

### 2.2. Clinical and Radiological Assessment

Data were collected through a comprehensive chart review that included the presence and extent of cholesteatoma, comorbid otitis media with effusion, the frequency of otorrhea episodes, and pretreatment infection status. Otorrhea episodes were identified based on information extracted from the medical records, including reports provided by the patients and/or their caregivers as well as documented otoscopic findings recorded during clinical visits. Bacteriological findings were obtained from otorrhea cultures performed preoperatively. Radiological evaluation was conducted using pretreatment computed tomography to assess the degree of mastoid pneumatization and the presence of aeration in the tympanic cavity based on the staging and classification criteria proposed by the Japan Otological Society [7]. Surgical records were reviewed to identify the need for ossicular reconstruction.

### 2.3. Audiometric Evaluation

Pretreatment audiometric values were primarily measured at the initial visit. Postoperative audiometric values were measured at least six months after surgery. The mean air conduction (AC) and BC thresholds were calculated as four-frequency averages at 0.5, 1, 2, and 3 kHz. Clinically significant hearing deterioration was defined as a mean BC threshold shift of ≥10 dB from the pretreatment to the postoperative period.

### 2.4. Bacteriological Evaluation

Pretreatment otorrhea samples were collected to identify causative organisms. To ensure that the samples accurately reflected the inflammatory environment of the middle ear, discharge was collected directly from the middle ear cavity during outpatient visits or immediately before surgery using sterile swabs, while carefully avoiding contamination from the external auditory canal. Discharge cultures were obtained as clinically indicated, and some patients underwent multiple culture examinations during the course of follow-up. Rather than restricting the analysis to a single preoperative culture result, all available culture results were included in the analysis. The culture results were categorized into three groups: (1) normal flora (e.g., skin commensals), (2) non-skin commensals (representing potential middle ear pathogens), and (3) no growth.

### 2.5. Statistical Analysis

Continuous variables were presented as medians with interquartile ranges (IQR), whereas categorical variables were expressed as numbers and percentages (*n*, %). Statistical analyses were conducted in three sequential steps to address the study objectives. To examine the relationship between the frequency of otorrhea and the severity of SNHL, a simple linear regression analysis was performed, and Pearson’s correlation coefficient (*r*) was calculated. The strength of the correlation was interpreted as follows: |*r*| < 0.2, very weak; 0.2 ≤ |*r*| < 0.3, weak; 0.3 ≤ |*r*| < 0.5, moderate; 0.5 ≤ |*r*| < 0.7, strong; and |*r*| ≥ 0.7, very strong. The coefficient of determination (*R*^2^), representing the proportion of variance in the dependent variable that was predictable from that of the independent variable, was used to evaluate the explanatory power of the model. To address potential confounding, multivariable linear regression models were constructed with BC threshold change as the dependent variable, including otorrhea frequency together with diagnosis (cholesteatoma vs. perforated otitis media) and the requirement of ossicular reconstruction as covariates. An otorrhea-frequency-by-diagnosis interaction term was tested to assess effect modification. Subgroup correlation analyses stratified by diagnosis and restricted to ears without ossicular reconstruction were also performed. Receiver operating characteristic (ROC) curve analysis was conducted to determine the predictive value of otorrhea frequency for clinically significant hearing loss (≥10 dB BC shift), and the optimal cutoff value was identified using the Youden Index. Additionally, to evaluate the impact of specific pathogens, the patients were divided into two groups: patients with non-skin commensals (representing potential middle ear pathogens) and those with either normal flora or no growth. The Mann–Whitney U test was used to compare the mean BC threshold changes between the two groups. Statistical significance was defined as *p* < 0.05. All statistical analyses were performed using JMP Student Edition v.19 (SAS Institute, Cary, NC, USA).

## 3. Results

### 3.1. Study Population

A total of 182 patients were initially screened, of whom 122 were excluded on the basis of the exclusion criteria (Figure 1). Finally, 60 patients (37 males, 23 females) were included in the analysis. The demographic and clinical characteristics of the patients are summarized in Table 1. The mean age at diagnosis was 9.7 years (range, 5–18 years, SD 3.4). A history of pretreatment infection was reported in 33 patients (55.0%). Ossicular reconstruction was performed in 22 patients (36.7%). The primary diagnoses were cholesteatoma in 46 patients (76.7%) and chronic perforated otitis media in 14 (23.3%). Among the patients with cholesteatoma, 15 (32.6%) were Stage I, 27 (58.7%) were Stage II, and 4 (8.7%) were Stage III, according to the Japan Otological Society criteria [7]. The median (IQR) duration from initial hearing assessment to surgical intervention was 144 days (45.0–189.3).

### 3.2. Correlation Between Otorrhea Frequency and Bone Conduction Deterioration

**Figure 2:**
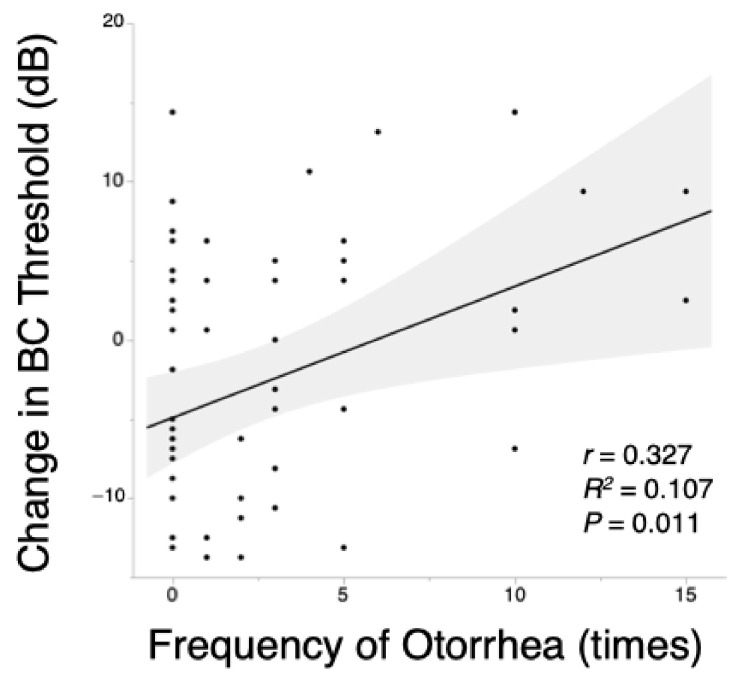
Correlation between the frequency of otorrhea episodes and pre- and postoperative changes in bone conduction thresholds. The scatter plot demonstrates a significant positive correlation between the frequency of otorrhea episodes and the deterioration of mean bone conduction thresholds (0.5, 1, 2, and 3 kHz) (*r* = 0.327, *R*^2^ = 0.107, *p* = 0.011). The solid line represents linear regression, and the shaded area indicates the 95% confidence intervals. BC, bone conduction; *r*, Pearson’s correlation coefficient; *R*^2^, coefficient of determination; *P*, *p*-value.

The frequency of otorrhea episodes showed a moderately positive correlation with the change in the mean BC threshold (*r* = 0.327, *p* = 0.011). In the simple linear regression model, this factor accounted for 10.7% of the variance in hearing deterioration (*R*^2^ = 0.107; Figure 2). Among all other clinical factors listed in Table 1—including age, sex, history of pretreatment infection, primary diagnosis, cholesteatoma stage, and tympanic membrane perforation size—none showed a significant association with the change in mean BC threshold (all *p* > 0.05).

### 3.3. Robustness of the Association Across Diagnosis and Surgical Manipulation

The association between otorrhea frequency and BC deterioration remained significant after adjustment for diagnosis (otorrhea frequency, β = 0.855, *p* = 0.014; diagnosis, *p* = 0.832), and the otorrhea-frequency-by-diagnosis interaction was non-significant (*p* = 0.756), indicating no effect modification by underlying disease. The direction of the association was consistent in both diagnostic subgroups (cholesteatoma, *n* = 46, *r* = 0.290; perforated otitis media, *n* = 14, r = 0.479). Similarly, otorrhea frequency remained a significant predictor after adjustment for the requirement of ossicular reconstruction (otorrhea frequency, β = 0.861, *p* = 0.010; ossicular reconstruction, *p* = 0.655), and the association remained directionally consistent among ears without ossicular manipulation (*n* = 22, r = 0.381, *p* = 0.080).

### 3.4. Predictive Value of Otorrhea Frequency for Clinically Significant Hearing Loss

**Figure 3:**
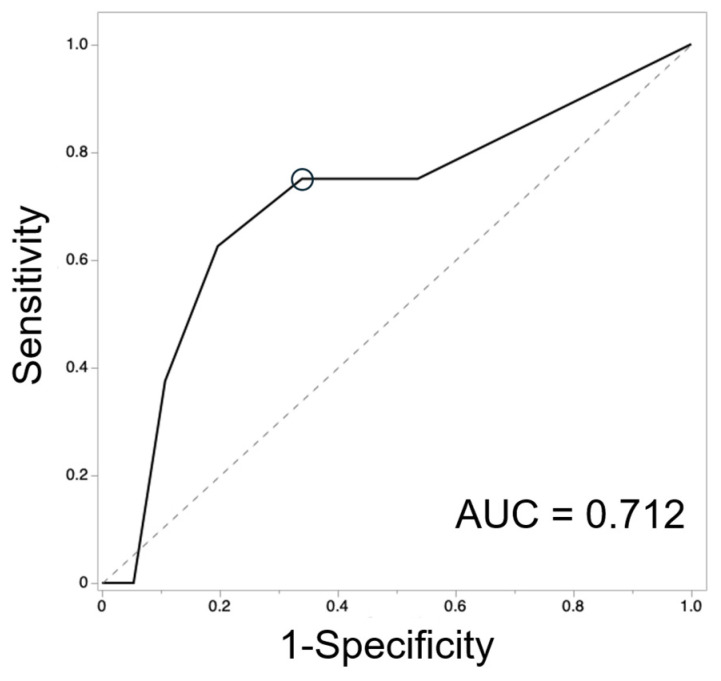
Receiver operating characteristic curve for predicting clinically significant sensorineural hearing loss. The ROC curve illustrates the performance of otorrhea frequency in predicting a mean BC threshold deterioration of ≥10 dB. The area under the curve was 0.712. The optimal cutoff point was identified as five episodes (sensitivity, 75.0%; specificity, 80.0%), as indicated by the Youden Index. BC, bone conduction; AUC, area under the curve; ROC, receiver operating characteristic.

ROC curve analysis identified five or more otorrhea episodes as the optimal cut-off for predicting a clinically significant BC shift of ≥10 dB (area under the curve [AUC] = 0.712; sensitivity, 75.0%; specificity, 80.0%; Figure 3).

### 3.5. Association Between Bacteriological Findings and BC Threshold Changes

The presence of potential pathogens did not significantly affect the hearing outcomes. No significant difference in mean BC threshold changes was observed between patients with non-skin commensals (*n* = 6) and those with normal flora or no growth (*n* = 54) (*p* = 0.721, Mann–Whitney U test).

## 4. Discussion

### 4.1. The Main Finding: Otorrhea Frequency as a Surgical Criterion

Surgical indications for pediatric COM have long lacked consensus. The present study demonstrated that the cumulative frequency of otorrhea episodes is a strong predictor of SNHL severity. We identified “five episodes of otorrhea” as a novel and objective quantitative threshold for predicting irreversible BC threshold shifts of ≥10 dB. Although traditional management often relies on the presence or absence of discharge, our findings suggest that the recurrence rate is a superior clinical metric for gauging inner ear risk. This objective “five-episode” red flag allows clinicians to move beyond subjective assessments and justify timely surgical intervention to safeguard cochlear function.

### 4.2. Interpretation and Comparison: Mechanism of Inner Ear Damage

The mechanism underlying SNHL in COM has been extensively debated. Although conductive loss is secondary to mechanical obstruction, sensorineural damage arises from the diffusion of inflammatory products across the round window membrane. In adults with COM, the prevalence of SNHL has been reported to be as high as 52% [8]. Recent evidence suggests that the primary driver of cochlear injury is not direct bacterial toxicity but rather the host’s immune response within the inner ear. Recurrent inflammation triggers a significant increase in the number of macrophages, particularly at the basal turn of the cochlea, leading to the destruction of outer hair cells [9]. In our analysis, recurrent otorrhea reflected the total duration of inflammatory exposure and served as an indirect index of the cumulative immune response. Our data show that these “toxic pulses” to the inner ear begin far earlier in the pediatric population than previously recognized.

### 4.3. Features of the Pediatric Population: Challenging the “Wait-And-See” Paradigm

Although the surgical management of pediatric COM is complicated by higher recurrence rates, often attributed to eustachian tube immaturity, delaying surgery based on age alone may allow irreversible cochlear injury to progress undetected. Consequently, many clinicians advocate delayed surgery; for instance, Neha et al. [10] suggested waiting until age 8, and Shinohara et al. [11] recommended waiting until age 10 years based on improved graft take-rates. However, Hunter et al. reported that children aged 3–5 years exhibited elevated high-frequency BC thresholds [4]. Because pediatric hearing is often difficult to measure accurately and inflammation frequently begins around the age of 1 year, inner ear damage likely precedes clinical detection. Even minor shifts in BC can significantly affect language acquisition and academic potential [3]; thus, a uniform age-based criterion may be counterproductive. We argue that otorrhea frequency, in addition to age, should help guide the timing of surgical intervention.

### 4.4. Clinical Implications: A Tool for Non-Specialists

Otorrhea is a visible and easily monitored symptom. The “five-episode” threshold is particularly useful because it can be utilized by pediatricians and primary care physicians with no specialized otological equipment or advanced knowledge. Children experiencing frequent otorrhea are at high risk of irreversible SNHL and should be prioritized for early surgical referral. By establishing this quantitative red flag, we can facilitate earlier intervention, bridge the gap between primary care and specialized otosurgery, and prevent lifelong social and developmental consequences associated with pediatric hearing loss.

### 4.5. Limitations

This study had several limitations that should be acknowledged. First, the inclusion of very young children may have affected the data quality, as the reliability of BC thresholds cannot always be fully guaranteed in this age group. Behavioral audiometry in preschoolers can be technically challenging, and even with techniques such as play audiometry, thresholds can be influenced by developmental maturity and concentration levels. Second, our cohort was predominantly composed of cholesteatoma (46/60), and although the association between otorrhea frequency and BC deterioration was consistent across diagnostic subgroups with no significant interaction, the relatively small number of perforated-otitis-media ears (*n* = 14) and of clinically significant BC shifts (*n* = 4) precluded a fully powered, diagnosis-stratified analysis. Confirmation in larger, multi-institutional cohorts is warranted. Third, although BC was selected specifically because it reflects cochlear function and is relatively insulated from intratympanic/ossicular manipulation, a procedure-related contribution to BC change—such as drilling-induced cochlear trauma or the extent of surgical dissection—cannot be entirely excluded in this retrospective design. Acoustic trauma from high-speed drilling and suction or mechanical manipulation of the ossicular chain are known iatrogenic factors that can cause threshold deterioration. Although we measured hearing at least six months postoperatively to ensure stability, the potential confounding effect of surgical intervention remains a consideration. Finally, the possibility cannot be excluded that treatments administered during the disease course, including systemic antibiotic therapy and topical ear-drop treatment for episodes of otorrhea, may have influenced the microbiological findings. Furthermore, the specific risks associated with different pathogens, such as Pseudomonas aeruginosa biofilms, require further exploration.

## 5. Conclusions

The frequency of otorrhea is a critical clinical indicator of the risk of SNHL in pediatric patients with COM. We propose five or more otorrhea episodes as the objective age-independent threshold for surgical referral. This metric provides a simple yet powerful tool for clinicians to protect inner ear function and optimize long-term developmental quality of life in children with chronic ear diseases.

## Figures and Tables

**Table 1:** Demographic features of enrolled patients.

No. of Patients	60
Age at diagnosis (year), mean (range)	9.7 (5–18)
Sex, *n* (%)	
Male	37 (61.7)
Female	23 (38.3)
Infection history, *n* (%)	
Yes	33 (55.0)
No	27 (45.0)
Diagnosis, *n* (%)	
Chronic perforated otitis media	14 (23.3)
Cholesteatomatous otitis media	46 (76.7)
Extension of cholesteatoma	
Stage I	15 (32.6)
Stage II	27 (58.7)
Stage III	4 (8.7)
Infection status, median (IQR)	
Total duration of otorrhea (days)	7.0 (7.0)
Frequency of otorrhea episodes	1 (3.0)
Concomitant otitis media with effusion	
Presence	17 (28.3)
Absence	43 (71.7)
Computed tomography, *n* (%)	
Degree of mastoid pneumatization ^1^	
Grade 0 (Sclerotic/None)	4 (6.7)
Grade 1 (Poor)	19 (31.7)
Grade 2 (Moderate)	24 (40.0)
Grade 3 (Good/Goodly pneumatized)	13 (21.7)
Aeration in the tympanic cavity	
Present	45 (75.0)
Absent	15 (25.0)
Results of otorrhea culture, *n* (%)	
Normal flora	16 (26.7)
Non-skin commensals ^2^	6 (10.0)
No growth	38 (63.3)
Pure-tone audiometry (dB), median (IQR)	
Pretreatment AC threshold	32.8 (26.7)
Pretreatment BC threshold	8.1 (9.8)
Postoperative AC threshold	25.9 (22.3)
Postoperative BC threshold	5.0 (9.1)
Details of surgery, *n* (%)	
Ossicular reconstruction	
Not required	38 (63.3)
Required	22 (36.7)

^1^ Categories based on the proposal of the Japanese Otological Society [7]. ^2^ Methicillin-resistant *Staphylococcus aureus*, *Pseudomonas aeruginosa*, *Haemophilus influenzae*, and *Enterococcus* spp. Thresholds were calculated as the 4-frequency mean of 0.5, 1, 2, and 3 kHz. AC, air conduction; BC, bone conduction; IQR, interquartile range.

## Data Availability

The data presented in this study are available upon request from the corresponding author. The data are not publicly available due to privacy and ethical restrictions.

## Abbreviations

The following abbreviations are used in this manuscript:

COM	Chronic otitis media
SNHL	Sensorineural hearing loss
ROC	Receiver operating characteristic
BC	Bone conduction
QOL	Quality of life
AC	Air conduction
IQR	Interquartile range
AUC	Area under the curve
